# Colorectal Cancer in a Patient With Multiple Myeloma: A Treatment Dilemma

**DOI:** 10.7759/cureus.12112

**Published:** 2020-12-16

**Authors:** Chung-ting J Kou, Joshua Romain, Devin R Broadwater, Taylor Barnett

**Affiliations:** 1 Internal Medicine, Brooke Army Medical Center, Fort Sam Houston, USA; 2 Hematology and Oncology, Brooke Army Medical Center, Fort Sam Houston, USA; 3 Pathology, Brooke Army Medical Center, Fort Sam Houston, USA

**Keywords:** multiple myeloma, secondary primary malignancy, colon adenocarcinoma, bisphosphonate, fluoropyrimidines

## Abstract

Multiple myeloma (MM) is a malignancy of plasma cells characterized by the clonal proliferation of plasma cells that produce monoclonal immunoglobulins. While typically considered to be incurable, advances in treatment options have led to remarkable improvements in survival for these patients. Accumulating evidence suggests an increased risk for the development of a secondary primary malignancy (SPM) in these patients, perhaps as a result of myeloma directed therapy or as an effect of their underlying disease process. Here we present a case of a patient who was diagnosed with an SPM while undergoing palliative treatment for multiple myeloma and a treatment approach.

## Introduction

Immunomodulatory agents have become an integral component of multiple myeloma treatment. As more data accumulate regarding the efficacy of these agents, we continue to learn more about their associated risks. Specifically, there are multiple reports suggesting an increased risk of the development of a secondary primary malignancy (SPM) among patients treated with these agents. Given iterative survival improvements seen with these new regimens, patients with multiple myeloma (MM) will continue to live longer, providing an increased time to develop these SPMs. As multiple myeloma is typically not considered to be curable (with the rare exception of allogeneic stem cell transplantation), providers will increasingly be presented with the dilemma of selecting an appropriate treatment regimen for patients with myeloma and SPM, where respective treatment options often have very little overlapping efficacy. Here we present the case of a patient who developed stage III colon adenocarcinoma while receiving induction therapy for myeloma and describe a potential treatment approach.

## Case presentation

**Figure 1:**
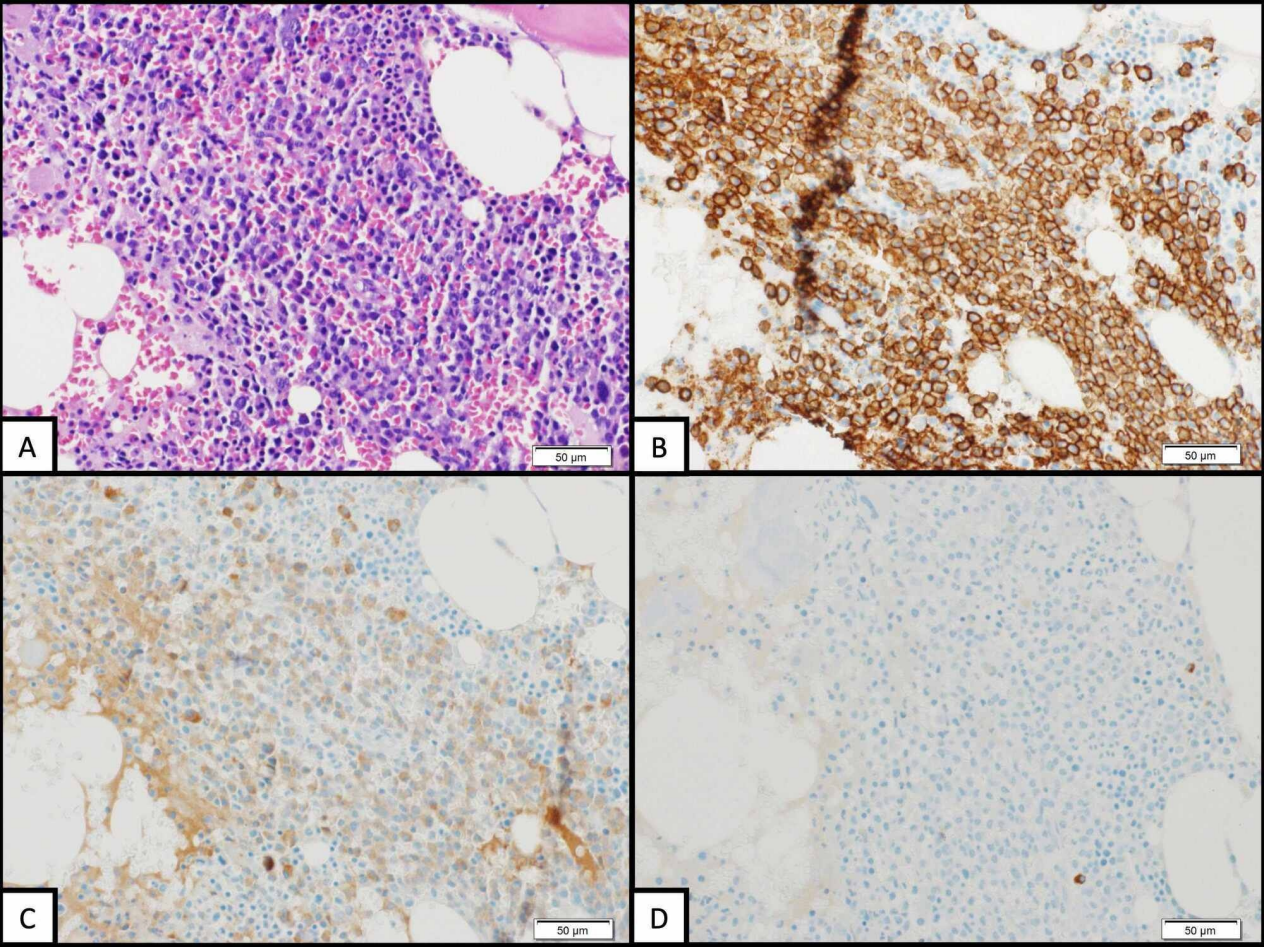
Bone marrow core biopsy histology (A) Bone marrow core biopsy histology at 200x magnification demonstrates hematoxylin and eosin (H&E) stained section showing hypercellular marrow with trilineage hematopoiesis with abundant small mature plasma cells. (B) CD138 immunostain highlights abundant plasma cells with membranous staining. Comparing Kappa immunostain (C) to Lambda immunostain (D) demonstrates dim kappa restricted plasma cells consistent with myeloma diagnosis.

A 73-year-old man with a past medical history significant for remote soft tissue sarcoma of the right upper extremity, treated with wide excision and adjuvant radiation, presented to the emergency department in December 2018 complaining of severe left-sided rib pain. Cross-sectional imaging was notable for diffuse osteolytic lesions throughout his visualized appendicular and axial skeleton. Serum protein electrophoresis (SPEP) demonstrated an M-spike of 2.28 g/dL (IgA kappa), with a kappa/lambda light chain ratio of 307, lactate dehydrogenase (LDH) of 148 U/L, β2-microglobulin of 1.6 mcg/mL, and albumin of 3.9 g/dL. Bone marrow biopsy (Figure 1) confirmed the diagnosis of multiple myeloma with 70% plasma cell involvement and del16q noted on fluorescence in situ hybridization (FISH). Based on the patient’s β2-microglobulin, albumin, LDH, and cytogenic on FISH, his multiple myeloma was staged as stage I per the revised international staging system (R-ISS). 

**Figure 2:**
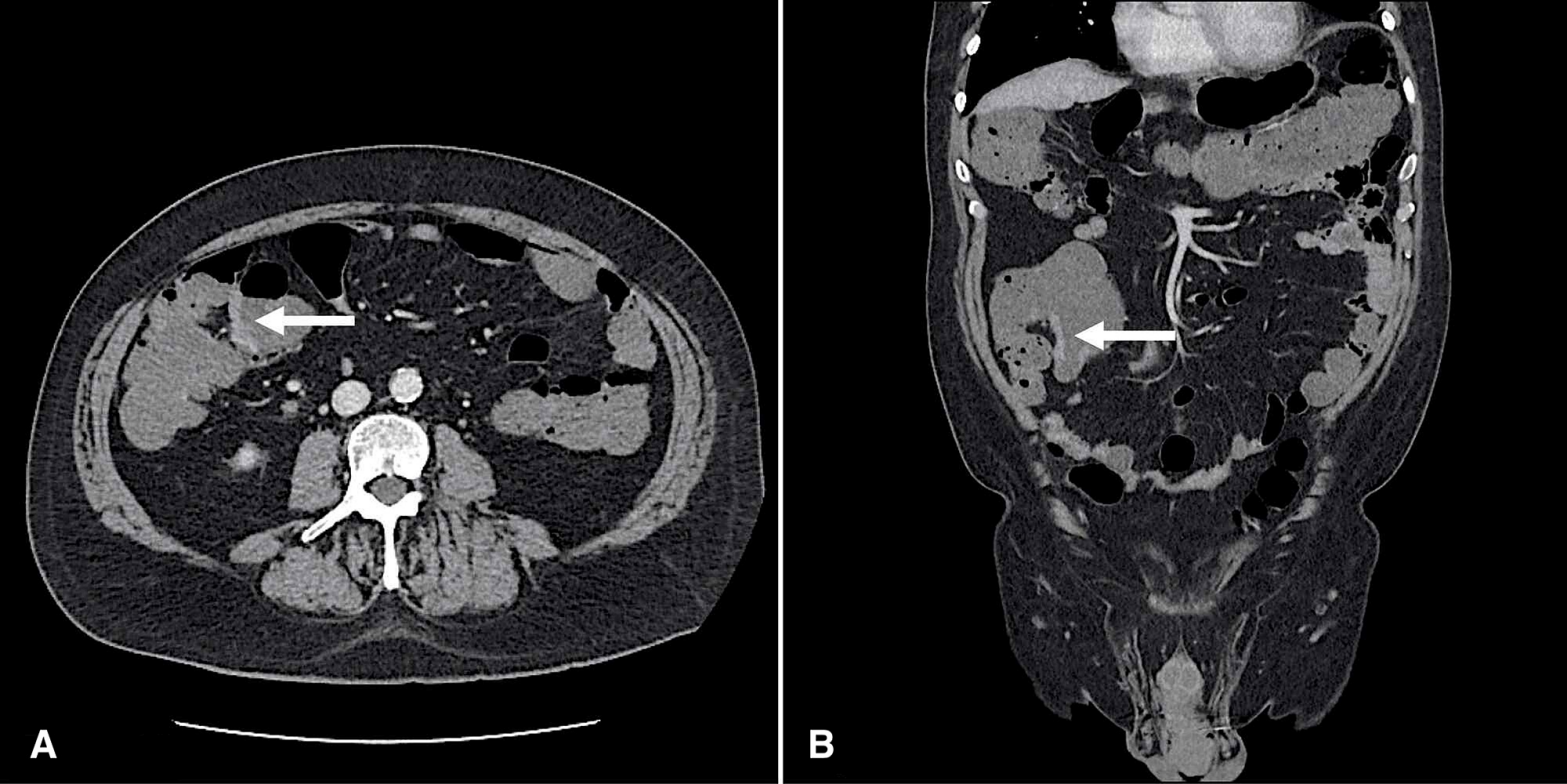
Computed tomography of the abdomen and pelvis with contrast Axial cross-section (A) and coronal cross-section (B) demonstrating focal asymmetric wall thickening and enhancement (white arrow) along the right lateral margin of the cecum measuring 5 mm in thickness concerning for potential colon cancer. The remainder of the gastrointestinal tract with normal wall thickness without any other surrounding inflammatory changes or visible paracolic adenopathy.

The patient was started on lenalidomide, bortezomib, dexamethasone regimen (RVD), and zoledronic acid. He achieved a partial response (PR) by International Myeloma Working Group (IMWG) response criteria in March 2019 following cycle three of RVD, with an M-spike of 1.05 g/dL. The patient was determined to be a good candidate for autologous transplant based on his performance score, lack of organ dysfunction, and underwent successful stem cell collection. Following cycle six of RVD, he remained in partial remission. He was transitioned to daratumumab, pomalidomide, and dexamethasone regimen (Dara-Pom-Dex) to achieve the deepest possible response prior to autologous stem cell transplant. Following cycle two of Dara-Pom-Dex, his M-spike had fallen to 0.27 g/dL. However, his treatment was held for abdominal pain and complaint of dark stools. A CT of the abdomen and pelvis demonstrated colonic thickening in the ascending colon (Figure 2), prompting biopsy via colonoscopy.

**Figure 3:**
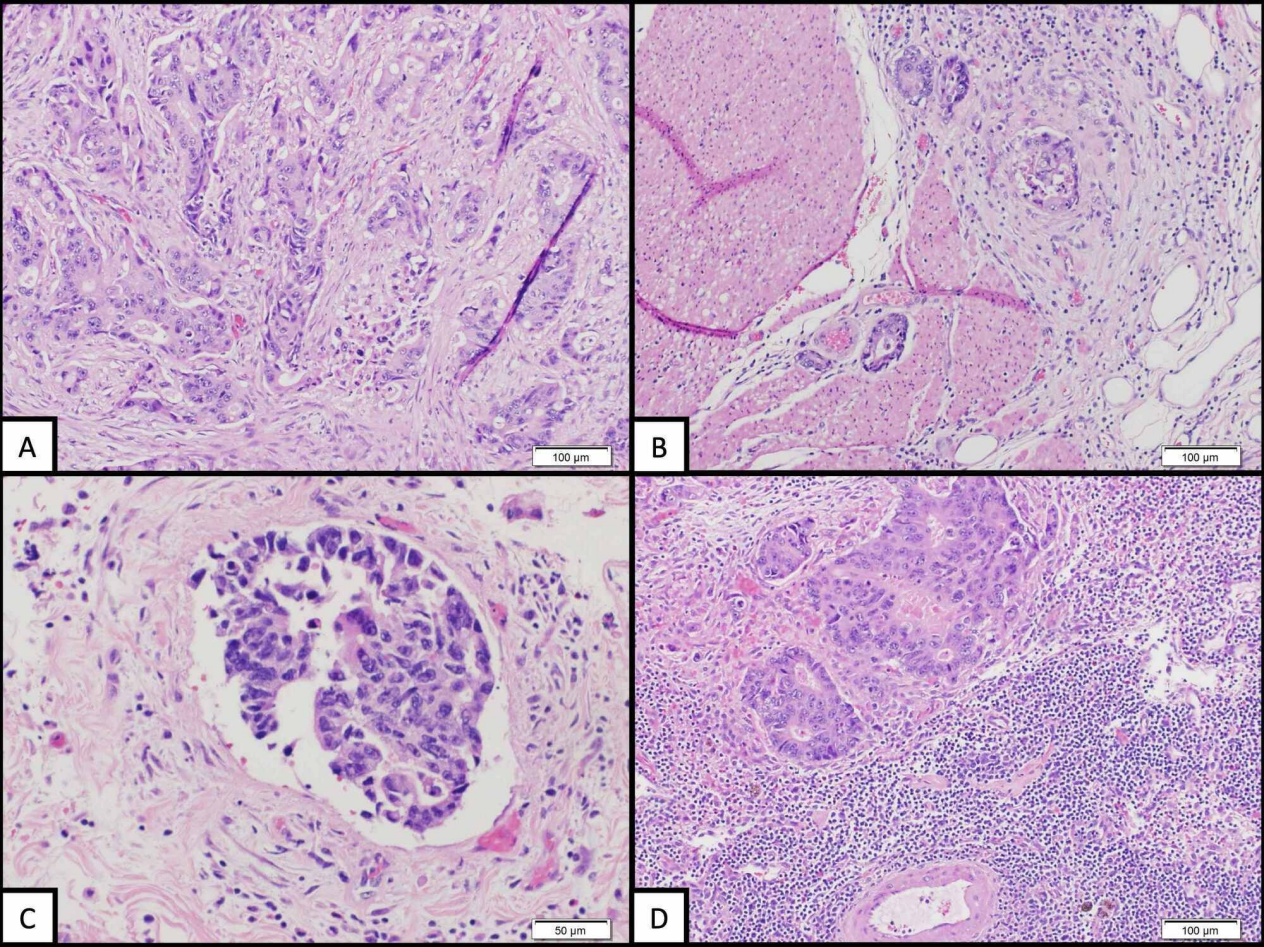
Histology of right hemicolectomy tissue Right hemicolectomy tissue H&E stained histology (parts A, B, D at 100x magnification, part C at 200x magnification) illustrates infiltrative tumor morphology that is moderate to poorly differentiated with stromal retraction (A), tumor invasion through the muscularis propria into pericolorectal adipose tissue (B), the lymphovascular invasion of tumor cells (C), and tumor deposits in a regional lymph node (D).

The colonoscopy showed a 4 cm cecal mass with biopsy confirming the diagnosis of moderately differentiated adenocarcinoma. Of note, the patient had been up to date with colon cancer screening with his last screening colonoscopy at age 70, which showed hyperplastic polyps in the cecum. He underwent successful robotic-assisted right hemicolectomy and was found to have stage IIIB (pT3N2aM0) colorectal cancer (Figure 3). Microsatellite instability analysis (MSI) immunochemistry showed intact staining for MLH1, MSH2, MSH6, and PMS2 within the tumor.

The patient was started on adjuvant capecitabine, with the continuation of bisphosphonate therapy, and completed six months of therapy. During this time, the patient’s myeloma was stable with an M-spike of 0.26 g/dL at the completion of capecitabine. His myeloma directed therapy with daratumumab, pomalidomide, and dexamethasone was subsequently resumed.

## Discussion

Advancements in the therapeutic management of multiple myeloma, including combination high-dose melphalan and autologous stem cell transplantation, as well as the introduction of novel immunomodulatory anti-myeloma agents and proteasome inhibitors, have increased the life expectancy of multiple myeloma patients. However, with prolonged survival, managing clinical conditions associated with increased survival, like SPM, becomes more clinically relevant. SPM primarily manifests as hematological malignancies with an overall seven-fold increase of acute myeloid leukemia (AML) following MM diagnosis [1]. However, solid SPMs also have been reported in a population study of 36,491 MM cases in the Surveillance Epidemiology and End Result (SEER) Program between 1973 and 2008 with a heterogeneous distribution of risk based on the specific solid subtype [1]. The association between MM treatment and SPM was first identified in 1996 by Govindarajan et al. with alkylating agents in high dose chemotherapy, particularly melphalan [2]. Additionally, Krishnan et al. identified an 11.2% risk of developing SPM 10 years after autologous hematopoietic stem cell transplantation [3]. With the advancement of immunomodulatory drugs, there appears to be an association between lenalidomide and SPM, particularly in patients who were also exposed to alkylating agents such as oral melphalan [3-6]. The cumulative five-year risk of solid SPM in newly diagnosed MM patient treated with lenalidomide was 3.1% (95% CI: 1.9-4.3) versus 1.4% (95% CI: 0.0-3.6) in patients who did not receive lenalidomide as demonstrated by a 2014 meta-analysis of seven randomized, controlled, phase III clinical trials [6]. The cumulative five-year risk of hematological SPM in the same meta-analysis was 3.8% (95% CI: 2.7-4.8) in newly diagnosed MM patients treated with lenalidomide versus 3.4% (95% CI: 1.5-5.2) in MM patients who did not receive lenalidomide [6]. Development of SPM is most likely multi-factorial, including MM treatment, tumor microenvironment, genetic, and environmental factors [7]. The mechanism of lenalidomide is complex, and its ability to promote SPM development remains unclear. Lenalidomide’s immunosuppressive effects on the tumor microenvironment may lead to the escape and/or growth of abnormal tumorigenic clones resulting in the development of an SPM [5]. In vitro studies have demonstrated that cereblon, a molecular target for lenalidomide, is a component of the E3 ubiquitin-ligase complex that is essential to nucleotide excision repair [8]. Facilitation of SPM may arise as lenalidomide impairs the nucleotide excision repair mechanisms after melphalan-induced DNA damage [8]. More data are needed to elucidate the phenomenon of SPM in the setting of multiple myeloma following novel anti-myeloma treatment. In the meantime, patients should be informed of the risk of SPM when presented with the benefit and risk of various MM treatment options.

The increased incidence of hematological SPM after MM treatment has been consistently demonstrated in large population-based studies [1,9,10]. For example, AML SPM has a reported standardized incidence ratio (SIR) of 6.51 (95% CI: 5.42-7.83) [1]. However, the relationship between MM treatment and solid tumor SPM is less well established. The SIR is decreased for breast and prostate SPM, while it is increased for urinary bladder cancer after stratification based on age, latency, and year of MM diagnosis based upon SEER data [1]. Colorectal cancer risk was increased by 50% five years after MM diagnosis. Similarly, a retrospective Japanese study also demonstrated a significantly increased risk of colorectal cancer following MM diagnosis between 1984 and 1994, which may be due to associated immunologic abnormalities between both diseases [11]. However, in a similar epidemiological study of German and Swedish MM patients, the SIR was increased for kidney and nervous system cancers with decreased SIR for colorectal cancers [9]. It is unclear if any one specific factor led to our patient developing colorectal SPM during his treatment regimen or if he simply developed primary colorectal cancer due to his age. However, it is important to note that our patient had a screening colonoscopy three years before his MM diagnosis, which only showed hyperplastic polyps, and previous screening colonoscopies were without polyps. The risk of specific subtype solid SPM after MM treatment remains heterogeneous based on patient epidemiology, latency, and time period of diagnosis. This complicates the development and standardization of cancer screening strategies in MM patients post treatment. More surveillance data regarding SPM is needed to help develop potential cancer screening strategies for MM patients.

Additionally, during the course of his adjuvant capecitabine therapy for colorectal cancer, all myeloma directed therapy was held (except for zoledronic acid), and the patient’s surrogate markers for myeloma activity remained stable. A review of available literature did not reveal any study that evaluates the activity of fluoropyrimidines in the treatment of MM. It remains unclear why this patient maintained stable disease over a six month period without the administration of myeloma directed therapy, except for zoledronic acid. MM is characterized by osteolytic bone disease due to increased osteoclast activity and reduced osteoblast function, which is the principal pathophysiologic basis for using bisphosphonates in this disease process [12-14]. The role of bisphosphonates in multiple myeloma is to reduce the number of vertebral and non-vertebral fractures and osteolytic lesions [15]. Currently, there are no published clinical trials evaluating the role of bisphosphonates as a disease-modifying agent in MM. However, in vitro concentration and time-dependent cytotoxic effects of bisphosphonates have been demonstrated previously [16]. In this study, cytotoxic effects were seen in four out of five tested myeloma cell lines, which was attributed to a combination of cytostasis and apoptosis-mediated cell death. Similar in vitro findings have been reported with pamidronate exposure resulting in altered nuclear morphology and fragmented DNA in both JJN-3 and HS sultan cells, suggesting apoptosis [17]. A review of published case reports revealed two patients with progressive myeloma who were treated with pamidronate, resulting in stabilization of immunoglobulin (Ig) levels in the first patient and a >50% reduction in immunoglobulin (Ig) levels in the second patient [18]. There is a potential that our patient’s M spike may have stabilized as a result of his continued zoledronic acid during treatment of his colorectal cancer. However, more research is needed to establish the efficacy of bisphosphonates or fluoropyrimidines as disease-modifying agents before these conclusions could be drawn.

## Conclusions

Accumulating evidence suggests an association between MM and the development of SPM, whether as a treatment effect or an effect of the underlying disease process. This patient successfully completed definitive surgical therapy and adjuvant systemic therapy for colorectal cancer while holding his myeloma directed therapy without any identifiable complication. A temporary period of active observation of MM represents one potential management paradigm for patients presenting with an SPM during the course of MM treatment. The continuation of his bisphosphonate may have provided some anti-myeloma effect, and we cannot exclude the possibility that fluoropyrimidines may have activity in this disease process. Further studies would be necessary to evaluate these possibilities. Furthermore, clinicians need to be mindful of potential SPM development in MM patients in the absence of standardized cancer screening guidelines for MM patients after exposure to immunomodulatory therapy or cytotoxic chemotherapy prior to autologous transplant.
